# Spectrum of Dystonia in Spinocerebellar Ataxia

**DOI:** 10.5334/tohm.1163

**Published:** 2026-05-29

**Authors:** Siva roja Yellaturi, Adreesh Mukherjee, Sanjay Pandey

**Affiliations:** 1 Department of Neurology and Stroke Medicine, Amrita Institute of Medical Sciences, Mata Amritanandamayi Marg Sector 88, Faridabad, Delhi National Capital Region, India; 2 Department of Neurology, Bangur Institute of Neurosciences, Institute of Post Graduate Medical Education and Research, Kolkata, India

**Keywords:** Spinocerebellar Ataxia, Dystonia, Cerebellum, Pathophysiology, Management

## Abstract

**Background:**

Spinocerebellar ataxias (SCAs) are a diverse group of inherited disorders characterized by progressive cerebellar dysfunction. Beyond the classic ataxic features, dystonia is an important manifestation across both common and uncommon SCA subtypes. The complete clinical spectrum, pathophysiology, and treatment of dystonia in SCA remain incompletely understood.

**Objectives:**

This review aims to summarize the current literature on dystonia in SCAs, outlining its prevalence, clinical presentations, underlying mechanisms, and therapeutic strategies.

**Methods:**

The authors conducted a systematic literature review in PubMed (up to October 2025) with various search terms related to dystonia and spinocerebellar ataxia, including specific SCA types. Full text articles were included in the review based on clinical relevance.

**Results:**

Dystonia has been observed in the common SCAs such as SCA1, 2, and 3, as well as several of the rarer SCA types. While focal dystonias such as cervical dystonia and task-specific dystonia (writer’s cramp) are frequent manifestations, generalized dystonias are also documented. Dystonia may follow the ataxia, or can be the presenting symptom itself. Dystonia in SCA results from dysfunction in the interconnected brain networks primarily involving the cerebellum, basal ganglia, thalamus, and sensorimotor cortex. An altered dopaminergic signalling may be present as well. Treatment responses to levodopa, anticholinergics, botulinum toxin, and deep brain stimulation vary widely, underscoring the need for individualized therapeutic approaches.

**Conclusions:**

Recognizing dystonia as a part of the SCA spectrum is important for timely diagnosis and management. Further studies are required to elucidate the mechanisms and explore the targeted interventions.

## Introduction

Autosomal dominant spinocerebellar ataxias (SCAs) are a diverse group of inherited disorders primarily characterized by a progressive cerebellar dysfunction. While ataxia is the hallmark sign, many patients also exhibit various movement disorders, and dystonia is recognized as a clinically important manifestation [1]. Dystonia may be the presenting symptom in SCA, or may develop later in the course of the disease alongside ataxia [2,3]. However, the dystonia is not always described in a systematic way across the different SCA subtypes, and the information is often incomplete regarding the clinical characteristics (such as onset and distribution), and the therapeutic response. Furthermore, the underlying mechanisms linking cerebellar degeneration to dystonia are complex and still under investigation. Treatment options are also evolving, from pharmacotherapeutic to surgical modalities, making a detailed understanding of dystonia in SCAs essential for optimizing patient care. In this review, the authors aim to provide an overview of dystonia in the spinocerebellar ataxias examining the prevalence and clinical patterns, along with a discussion on the pathophysiology and the available treatment modalities.

## Methods

**Figure 1:**
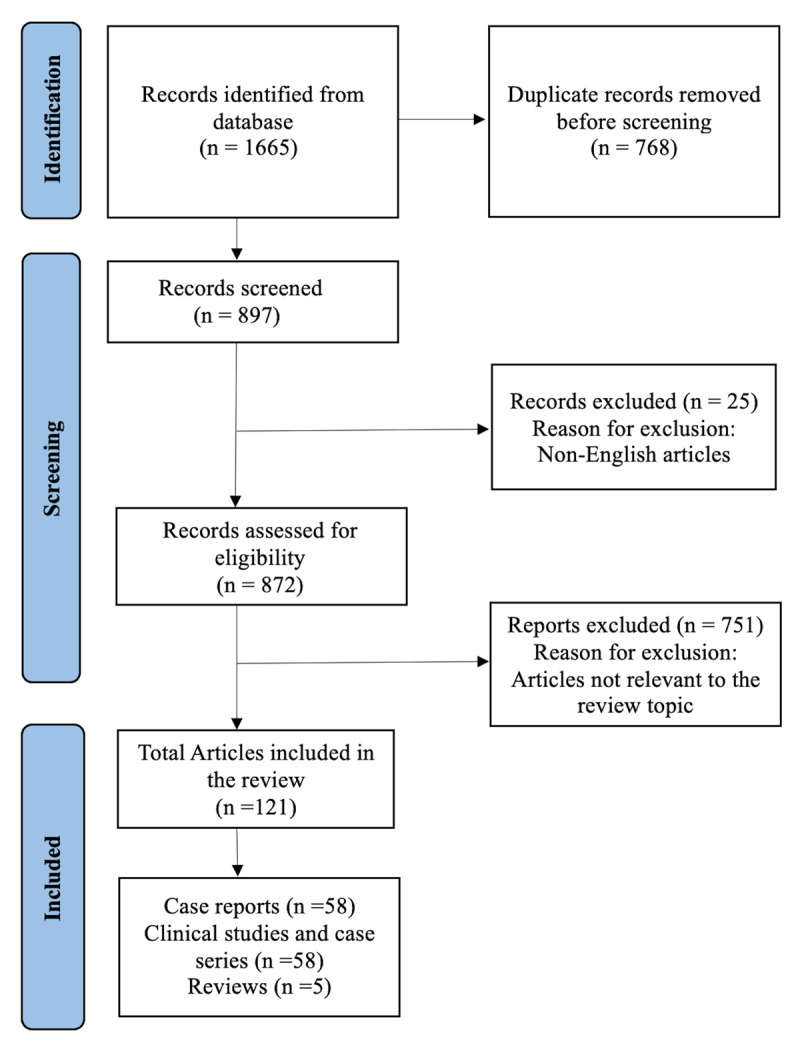
Study selection flowchart for the literature review of dystonia in Spinocerebellar ataxia.

A systematic literature search was conducted on PubMed database in October 2025 to explore the association between dystonia and SCA. The search strategy involved combinations of terms such as “dystonia” and “spinocerebellar ataxia,” “dystonia” and “SCA,” “dystonia” and specific SCA types (1–50), as well as “movement disorder(s)” with “spinocerebellar ataxia,” yielding a total of 1665 articles. After the removal of duplicate records, articles published in English were screened for inclusion based on clinical details and relevance to the research question. Studies meeting eligibility criteria underwent further review and were included in the final selection (Figure 1). Dystonia is also present in certain autosomal recessive cerebellar ataxias [4]. However, it is beyond the scope of this review, as the authors have included the autosomal dominant SCAs here.

Dystonia is characterized by sustained or intermittent, patterned and repetitive abnormal movements and/or postures, which may also be tremulous or jerky [5]. According to the 2025 dystonia classification [5], it was categorized into focal, segmental, multifocal, and generalized types, with distribution across seven body regions- upper face, lower face (including jaw, mouth, and tongue), neck (including shoulders), larynx, upper limbs (including upper and lower arms and hands but not shoulders), trunk, and lower limbs (including upper and lower legs and feet).

### SCA1

In an Indian cohort comprising 85 patients with SCA types 1–3, extrapyramidal signs were least prevalent in SCA1, with dystonia observed in only 12.5% of SCA1 cases compared to 17.9% in SCA2 and 17.6% in SCA3, though these differences were not statistically significant [6]. Other cohorts similarly reflect the relative rarity of dystonia in SCA1, with a Serbian study reporting dystonia in only 9.1% of SCA1 patients compared to 44.4% in SCA2 [7], and a Thai study finding severe generalized dystonia in 4.8% of SCA1 patients (1/21) [8]. Dystonia in SCA1 can occasionally present as an initial manifestation preceding ataxia [9]. The size of the expanded CAG repeat length does not show a direct correlation with the presence of extrapyramidal signs or dystonia, as even modest repeat expansions may be associated with dystonia [6,9]. While focal (such as cervical) or generalized dystonia is typical in SCA1, it may rarely present as a task-specific dystonia such as writer’s cramp [10,11]. Additionally, a patient with generalized dystonia and recurrent dystonic crises showed a remarkable response to Globus pallidus internus (GPi) deep brain stimulation [12]. Overall, dystonia in SCA1 is relatively infrequent but exhibits considerable clinical heterogeneity.

### SCA2

In SCA2, dystonia exhibits a variable prevalence across different populations, with cohorts from Brazil, India, Taiwan, and Martinique reporting less than 15% of patients affected [13,14,15,16,17,18]. Conversely, higher frequencies ranging from 44.4% to 66.6% have been observed in Serbia and certain hereditary ataxia cohorts [7,19]. Notably, dystonia in SCA2 has been associated with longer expanded CAG repeat lengths at the *ATXN2* gene, although no clear relationship with age at onset or disease duration has been demonstrated [13]. The commonest form of dystonia in SCA2 is focal dystonia such as cervical dystonia, which may precede the onset of ataxia by several years [16,20,21,22]. Importantly, the cervical dystonia may be levodopa-responsive [23]. Oromandibular and other lower cranial dystonias, including jaw-opening and lingual protrusion, are additional clinical presentations [2,24] that can be refractory to dopaminergic and anticholinergic treatment [25]. Spasmodic dysphonia has been reported in Irish SCA2 families [26]. Limb dystonia including, the task-specific writer’s cramp, and speech-induced mouth dystonia, may also manifest in SCA2 [7,27,28,29,30]. Dystonia-parkinsonism phenotypes have been documented in the Canadian and Chinese SCA2 populations, where dystonia may precede or accompany parkinsonian symptoms [31,32]. Rare clinical variants include neonatal-onset SCA2 presenting with encephalopathy and prominent early dystonia with profound dystonic jerks, described as an exceptional phenotype [33].

### SCA3

The prevalence rates of dystonia in SCA3 are variable, with reported rates ranging from approximately 2.6% to 50% in different cohorts [6,8,14,17,34,35,36,37,38]. Compared to other SCA subtypes, dystonia is more commonly and distinctly associated with SCA3 [16,19,34,37,39]. Clinically, dystonia frequently presents as focal forms such as blepharospasm, oromandibular dystonia, cervical dystonia, writer’s cramp, and segmental dystonias [15,40,41,42,43]. Some cases exhibit dopamineresponsive dystonia or dystonia with diurnal fluctuation [44,45,46], and focal dystonia can precede classic ataxic symptoms by years [42,45,47], sometimes presenting as the sole clinical manifestation [41,42,48]. In one report, SCA3 presented as an isolated orofacial dystonia resembling Meige’s syndrome without cerebellar signs [48]. Another study described tongue dystonia in a childhoodonset SCA3 case [49]. Severe truncal dystonia resembling Pisa syndrome has also been reported as a rare phenotype in SCA3 [50]. The presence of dystonia in SCA3 often correlates with earlier disease onset and larger CAG repeat expansions in some cohorts [14,51], although correlations with disease severity or progression are inconsistent across studies [40]. In a large series of SCA patients, dystonia was most common in SCA3 (24.6%), followed by SCA2 (18%), SCA1 (12%), and SCA6 (8.6%) [52]. Moreover, SCA3 with dystonia had an earlier onset and longer CAG repeat numbers [52].

### SCA5, SCA6

Dystonia was reported in one of the three SCA5 patients in a Canadian study, although the specific body region affected by dystonia was not detailed [53]. SCA6 is generally characterized by a relatively uniform clinical phenotype, predominantly manifested as cerebellar ataxia without frequent extrapyramidal features. Although rare, dystonia is increasingly recognized as a potentially disabling clinical feature in SCA6. Cases reported include levodopa-responsive focal dystonia localized to the limbs [53], painful fixed dystonia of the arm, and bilateral dystonic foot inversion, with variable responses to treatments such as botulinum toxin, levodopa, and surgical interventions [54]. Focal dystonias, such as writer’s cramp, have been documented, occasionally preceding the onset of cerebellar ataxia by several years [55,56]. Family studies reveal phenotypic heterogeneity, with some affected members exhibiting writer’s cramp along with ataxia symptoms [57]. Focal hand dystonia before ataxia onset has also been described in Indian [16] and Korean patients [35]. Other reported focal dystonias include cervical dystonia accompanying early dysphagia and ophthalmoparesis in elderly patients [58], and lingual dystonia observed in siblings with concurrent cerebellar signs [59]. In a prospective study of hereditary ataxias, dystonia was documented in one of four genetically confirmed SCA6 patients, presenting as focal dystonia of the upper limb with accompanying postural tremor [60]. This indicates that dystonia may be present even in a supposedly “pure” SCA such as SCA6.

### SCA7

Dystonia in SCA7 is uncommon but shows diverse presentations, ranging from focal cranial forms to segmental and generalized patterns. It was reported in 2/20 patients (10%) as generalized dystonia in a Brazilian cohort [16] and in 2/4 patients as craniocervical dystonia in a large Chinese family [61]. It was also observed in 1/3 patients (33.3%) with spasticity in a Canadian multi-center study [53] and in 1/3 patients (33.3%) with segmental dystonia in the Portuguese cohort [19]. Brazilian data further documented focal blepharospasm in 1/7 patients (14.3%) [17] and dystonia without a subtype in 1/5 patients (20%) [37].

### SCA8, SCA10, SCA11

A patient of SCA8 developed prominent oromandibular and lingual dystonia alongside the characteristic features of adult-onset ataxia [62]. Additionally, a genetic screening study of Greek patients with Huntington’s disease phenocopies identified a patient with an SCA8 expansion who, besides the classic triad of chorea, psychiatric disturbance, and cognitive decline, also exhibited upper limb dystonia and oculomotor apraxia [63]. Another patient of SCA8 presented with levodopa-responsive parkinsonism and dystonia [64]. One member in an Argentinian family with SCA10 exhibited cervical dystonia [65]. Dystonia was reported in a single patient with SCA11 who exhibited laterocollis and upper limb tremor [66].

### SCA12

SCA12 exhibits diverse neurological symptoms exemplified by the presence of tremor [67]. Dystonia is also common, as shown in an Indian study where hand dystonia was present in 14 of 21 SCA12 patients, along with a case of cervical dystonia. Dystonia alongside the tremor can aid in the diagnosis of SCA12, and the tremor itself may have a dystonic component [68]. A study identified focal dystonia in three out of 37 genetically proven SCA12 patients, with cervical dystonia in two patients and Meige’s syndrome in another [16]. Similarly, in a Canadian cohort of rare spinocerebellar ataxia subtypes, one SCA12 patient of Indian/Punjabi origin developed dystonia in addition to parkinsonism, tremor, cerebellar ataxia, and sensory changes [69]. Several case reports further illustrate dystonia as an early or predominant feature in SCA12. A woman of Indian origin with SCA12 initially developed postpartum action tremor in her right arm, which later spread to the left arm, face, neck, and voice, manifesting as dystonic tremor and spasmodic dysphonia before the onset of ataxia [70]. This progressed with the development of tongue protrusion and feeding dystonia, causing feeding difficulties and weight loss [71]. Another SCA12 patient also manifested spasmodic dysphonia, followed by dystonic tremor and later ataxia [72]. Interestingly, O’Hearn et al., in their original description of an American family of German descent with SCA12, documented lower extremity dystonia in two affected individuals, which had begun in early childhood [73].

### SCA13, SCA14

In a Portuguese hereditary cerebellar ataxia cohort, segmental dystonia was identified in one out of four total SCA13 patients [19]. Dystonia in SCA14 manifests usually as writer’s cramp or cervical dystonia, and may be accompanied by a dystonic tremor (of the limb, head, or trunk), myoclonus, or other movement disorders such as parkinsonism and chorea [19,74,75,76,77,78,79,80,81,82,83]. A large multicenter study in individuals of European ancestry identified dystonia in only 8% of SCA14 cases, with one patient presenting writer’s cramp and another showing a complex phenotype including laterocollis and limb dystonia alongside progressive cerebellar ataxia [74]. However, two German cohorts reported a higher prevalence of dystonia (32–55%) [75,76]. Interestingly, in SCA14, writer’s cramp may be the initial symptom and precede the onset of ataxia by several years [77,78]. Foncke et al. reported a SCA14 patient with mild cervical dystonia, multifocal myoclonus, and an irregular trunk tremor. This trunk tremor was position-dependent, disappearing on lying down, consistent with a dystonic tremor rather than a classic cerebellar tremor. This particular phenotype makes SCA14 an important diagnostic consideration in patients who test negative for DYT11 and show cerebellar atrophy on Magnetic Resonance Imaging (MRI) [81].

### SCA17

Dystonia is a recognized clinical feature in SCA17, particularly common in individuals with CAG/CAA repeat sizes of 50–60 in the TATA-binding protein (TBP) gene [84]. In an Italian study, dystonia was present in approximately 53% (8/15) of SCA17 patients [85]. In SCA17, dystonia may coexist with chorea, parkinsonism, or rarely myoclonus [85,86,87]. The dystonia in SCA17 may be focal, affecting the limbs and neck [86,87,88], or may progress into a generalised dystonia [89]. A German SCA17 family reported focal dystonia as the presenting symptom in all three affected members, preceding cerebellar ataxia, with all three carrying TATA-binding protein (TBP) repeat expansions in the range of 53 to 55 repeats [90]. Notably, one case progressed to severe generalized dystonia exacerbated by action and emotional stress, highlighting dystonia as a potentially disabling manifestation of the disease [89]. In contrast, an Italian study of five SCA17 patients found dystonia to be rare and not a prominent feature; only one individual exhibited isolated mild hand dystonia early in the disease course, with normal dopamine transporter (DAT) binding [91]. In a SCA17 family with Huntington disease like phenotype, focal dystonia was reported in one of five affected members, although generalized chorea, cognitive decline, and gait imbalance predominated in most patients [92]. Another SCA17 patient presented with foot dystonia and spasticity [64]. A Brazilian patient with a small CAG/CAA expansion presented a complex movement disorder phenotype that included prominent upper limb, truncal, and cervical dystonia in addition to ataxia, parkinsonism, chorea, and cognitive symptoms [93]. This demonstrates that dystonia can be prominent even with small repeat expansions in SCA17.

### SCA19/22

The first case of dystonia in SCA19/22 was described in a Japanese patient who exhibited intellectual disability, early-onset cerebellar ataxia, myoclonus, and a right-sided upper extremity dystonia [94]. An Italian SCA19/22 patient presented with a neurodevelopmental disorder and prominent dystonia [95]. The dystonia began in adolescence with cervical and upper limb involvement, progressed to oromandibular dystonia and a persistent head tremor by his twenties, with marked dystonia evident at age 37 [95]. Patients in a Han Chinese cohort showed notable phenotypic heterogeneity, including cognitive impairment and various movement disorders, such as dystonia affecting both upper and lower limbs, or foot dystonia while walking [96]. In contrast, among the six Latin American SCA19 patients described in another study, only the proband developed oromandibular dystonia and dystonic posturing of both hands [97].

### SCA21

A 56-year-old Brazilian woman of remote Italian descent with SCA21 initially presented with upper limb and head tremor, which was misdiagnosed as essential tremor, but later developed cerebellar ataxia and focal hand dystonia [98]. A study on six members of a French family with SCA21 reported early-onset, prominent hand dystonic tremor, often accompanied by multifocal or generalized dystonia, including writer’s cramp in all the patients [99]. In the majority, dystonic tremor preceded or overshadowed mild ataxia, highlighting that dystonia, especially in the form of dystonic tremor, can be the dominant and initial clinical manifestation of SCA21 [99]. Dystonia was a prominent feature in three German patients with SCA21, who manifested an action dystonia or a myoclonus-dystonia phenotype [100]. A prospective study of 193 patients with hereditary cerebellar ataxia included two patients with SCA21, one of whom presented with segmental dystonia along with tremor [19]. Additionally, a 17-year-old girl with SCA21 from India showed prominent cervical and upper limb dystonia with dystonic head tremor and superimposed myoclonus in the form of a myoclonus-dystonia syndrome [101]. Her dystonia improved modestly with levodopa therapy.

### SCA28

In SCA28, dystonia has emerged as a notable though rare clinical feature. An Indian patient with SCA28 exhibited predominant generalized dystonia alongside the classical signs of cerebellar ataxia and external ophthalmoplegia [102]. Other reports on SCA28 also noted dystonia [53,103], including a case of generalized dystonia coupled with spasmodic dysphonia [103].

### SCA29, SCA34

In a study of hereditary cerebellar ataxias, dystonia was observed in two out of three patients (66.7%) with SCA29, predominantly presenting with a segmental pattern [19]. Another patient with SCA29 presented with prominent cervical dystonia in the form of retrocollis and mild ataxia [104]. A hospital-based, prospective, observational study of movement disorders in hereditary cerebellar ataxia found 1 patient of SCA34 with segmental dystonia [19].

### SCA35

In a Canadian cohort, dystonia was observed in only one SCA35 patient, and this individual also displayed other neurological findings such as gaze palsy, parkinsonism, sensory changes, and cognitive decline [53]. Dystonia may manifest as an isolated, treatment-resistant dystonic hand tremor without overt cerebellar signs [105]. Additionally, cervical dystonia has presented as a prominent feature in a pediatric case, occurring alongside dysarthria and intentional hand tremor [106]. In a foundational study identifying TGM6 as the causative gene for SCA35, the original Chinese family included four affected members who had spasmodic torticollis: two of these demonstrated both hand tremor and cervical dystonia, while the other two exhibited only cervical dystonia without accompanying tremor [107].

### SCA36

In a study from Eastern Spain, 5 out of 37 individuals with SCA36 exhibited dystonia, in the form of cervical dystonia, and asymmetric upper limb dystonic posturing and tremor [108]. Two Japanese siblings with SCA36 manifested cervical dystonia, and it was the initial symptom in one of them [109]. Another Japanese patient with SCA36 developed oromandibular dystonia with difficulty in mouth closure and eating alongside cerebellar ataxia and mild cerebellar atrophy, without evidence of parkinsonism or motor neuron involvement [110].

### SCA44, SCA48, SCA49, SCA50

In a prospective cohort study of hereditary cerebellar ataxia patients, dystonia was observed in a single individual with SCA44, presenting with a segmental distribution [19]. In two Italian families of SCA48, three out of eight patients demonstrated dystonia, presenting alongside ataxia, cognitive-psychiatric symptoms, chorea, and parkinsonism [111]. An Italian multicentre study found dystonia in five of eleven SCA48 patients, typically involving the cervical region (retrocollis or anterocollis), upper limbs, or appearing as blepharospasm with head tremor [112]. In these cases, dystonia frequently coexisted with chorea and parkinsonism, indicative of a Huntington-like disorder [112]. Other reports have also documented dystonia in SCA48, including a severe jaw-opening oromandibular dystonia [113] and mild facial and cervical dystonia [114]. Dystonia in SCA49 was described as a prominent feature in an Indian patient with cervical dystonia and dystonic head tremor in addition to ataxia and hyperreflexia [115]. In a family of SCA50 (*NPTX1*), the proband developed a generalized, appendicular dystonia along with postural limb tremor, and her son manifested dystonia of the lower limbs [61]. Interestingly, the brain MRI showed iron accumulation in basal ganglia and dentate nuclei.

**Figure 2:**
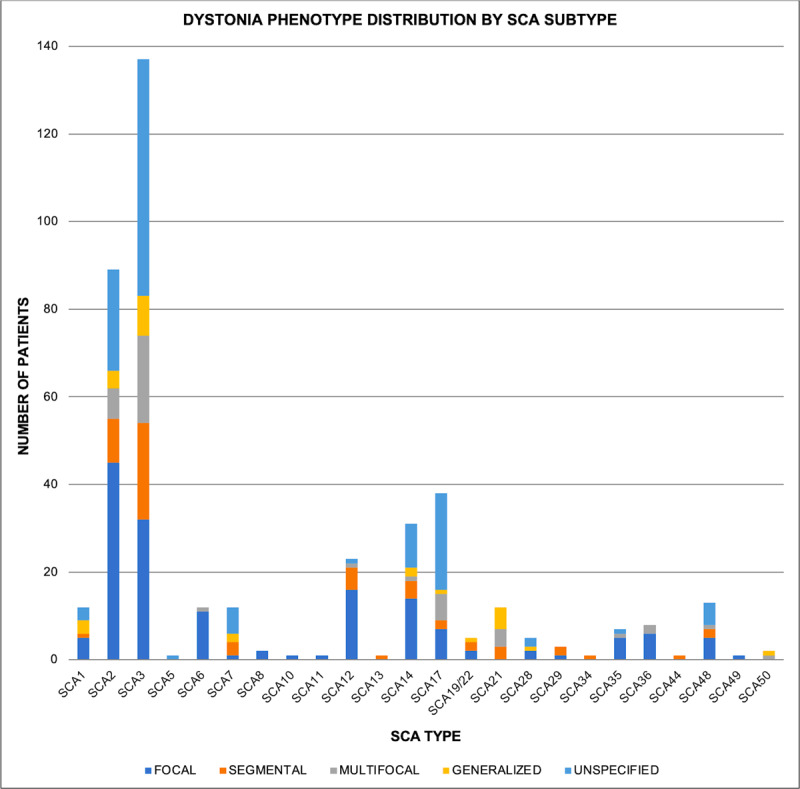
Dystonia phenotype distribution in the Spinocerebellar ataxia subtypes.

**Figure 3:**
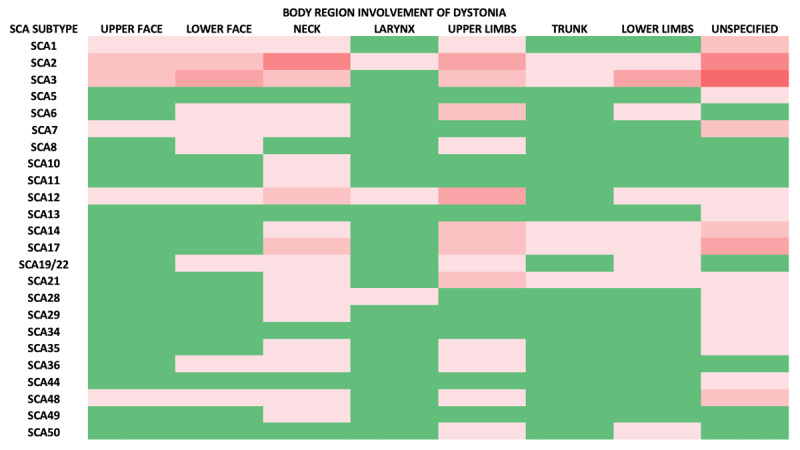
Heatmap showing the frequency of body region involvement in the Spinocerebellar ataxia subtypes (Green- minimum involvement, Red- involvement in increasing order).

Thus, to summarize the clinical findings, the dystonia in SCA manifests in various forms such as focal, segmental, multifocal, and generalized (Figure 2), and the distribution varies across the seven body regions of the upper and lower face, neck, larynx, upper and lower limbs, and trunk (Figure 3). The dystonia in SCA may be task-specific, such as writer’s cramp, and may also be associated with other movement disorders (Table 1). Dystonia frequently accompanies the ataxia and may be the predominant or even the initial manifestation.

**Table 1:** Salient features of dystonia in different Spinocerebellar ataxia types.


SCA SUBTYPE	SALIENT FEATURES

SCA1	• Focal (common- cervical) >generalised>segmental • Task specific writer’s cramp • Generalised dystonic crises • Good response to botulinum toxin

SCA2	• Cervical dystonia -most common • Lower cranial dystonia • Dystonia-parkinsonism • Levodopa responsive dystonia • Task specific writer’s cramp • Neonatal dystonia • Spasmodic dysphonia

SCA3	• Younger age of onset • Lower face dystonia-common • Facial grimacing • Task specific writer’s cramp • Levodopa responsive dystonia • Severe truncal dystonia • Early onset tongue dystonia

SCA5	• Unspecified

SCA6	• Focal dystonia-common • Levodopa responsive dystonia • Tongue dystonia • Disabling dystonia • Task specific writer’s cramp

SCA7	• Craniocervical-common

SCA8	• Focal dystonia • Oromandibular dystonia • Limb dystonia

SCA10	• Cervical dystonia

SCA11	• Cervical dystonia

SCA12	• Dystonic tremor • Spasmodic dysphonia • Focal hand dystonia • Feeding dystonia • Early childhood onset also reported

SCA13	• Segmental dystonia

SCA14	• Task specific writer’s cramp • Dystonic tremor • Dystonia-myoclonus • Dystonic trunk tremor

SCA17	• Dystonia-chorea • Dystonia-parkinsonism • Focal dystonia-common

SCA19/22	• Limb dystonia • Oromandibular dystonia • Dystonic tremor • Dystonia-myoclonus

SCA21	• Dystonic tremor • Focal hand dystonia • Task specific writer’s cramp • Myoclonus-dystonia • Levodopa responsive dystonia

SCA28	• Generalised dystonia • Spasmodic dysphonia

SCA29	• Cervical dystonia • Segmental dystonia

SCA34	• Segmental dystonia

SCA35	• Dystonic tremor • Cervical dystonia • Good response to botulinum toxin

SCA36	• Dystonic tremor • Cervical dystonia • Oromandibular dystonia

SCA44	• Segmental dystonia

SCA48	• Chorea-dystonia • Jaw-opening oromandibular dystonia • Cervical dystonia

SCA49	• Cervical dystonia

SCA50	• Appendicular dystonia


SCA- Spinocerebellar Ataxia, DBS- Deep Brain Stimulation, GPi- Globus Pallidus internus.

## Pathophysiology of Dystonia in Spinocerebellar Ataxia

The multifarious presentation of dystonia in SCA, along with the non-uniform therapeutic response (such as levodopa responsiveness), suggests that diverse pathways are possibly involved. Cerebellar dysfunction is pervasive in the SCAs, as evidenced by both structural and functional neuroimaging. Additional involvement may be present in the thalamus (ventral lateral and somatosensory nuclei), basal ganglia, nigrostriatal dopaminergic system, and the cerebral cortices [116], which are known to be associated with dystonia.

### Cortical dysfunction

In a SCA1 patient with cervical dystonia, hypermetabolism on Fludeoxyglucose-18 Positron Emission Tomography (FDG-PET) was present in the putamen and sensorimotor cortex, which normalized after botulinum toxin injection [11]. In a series of 21 SCA3 patients with dystonia, MRI volumetric analysis revealed atrophy of the precentral and paracentral cortices [117]. FDG-PET hypometabolism in the striatum and the parietal lobe, along with atrophy of the cerebellum and caudate nucleus on MRI volumetry, was present in a patient of SCA17 with dystonia [118]. A study utilizing transcranial magnetic stimulation demonstrated a reduction of the short interval intracortical inhibition (SICI) in SCA14 patients with dystonia [76]. However, in a patient of SCA3 where significant cerebellar atrophy was seen on 7-T MRI and cerebellar hypometabolism on FDG-PET, the cerebral cortex showed normal metabolism [119]. Although reduced intracortical inhibition is commonly present in dystonia, it appears that the intrinsic abnormality lies in the basal ganglia and the cerebellum, which in turn modulate the cortical activity [120]. Dystonia is also accompanied by deranged sensorimotor integration, and enlarged cortical somatosensory receptive fields with impaired intracortical inhibition and aberrant somatotopy [121,122,123]. However, the cortical changes may be secondary to the abnormal connectivity from subcortical structures such as the basal ganglia and thalamus [121].

### Subcortical dysfunction

The basal ganglia are considered to be a primary contributor to the pathophysiology of dystonia. Basal ganglia degeneration was noted on MRI in three patients of SCA3 with dystonia [124]. Caudate atrophy and striatal hypometabolism were seen in a SCA17 patient with dystonia [118]. As some of these patients showed a good response to levodopa, the dopaminergic function was investigated. A SCA2 patient with orolingual dystonia and unremarkable basal ganglia on MRI showed a decreased presynaptic dopamine transporter binding [25]. Analysis by a Single-Photon Emission Computed Tomography (SPECT) study on SCA2 patients with cervical dystonia noted reduced striatal binding of ^123^I-β-CIT and ^123^I-IBZM denoting, the impairment of both pre-synaptic Dopamine Transporter (DAT) binding and post-synaptic D2 receptors, respectively [22]. However, D2 receptor function was normal (^11^C-raclopride binding) in other SCA2 patients presenting with parkinsonism and focal dystonia despite a reduced striatal ^18^F-fluoro-L-dopa uptake [125]. These patients had improvement with levodopa [125]. Other reports have also shown levodopa-responsive dystonia in SCA2 [23] and SCA3 [45,46]. Interestingly, Lewy body pathology has been demonstrated in two SCA2 patients with parkinsonism [126]. Even SCA6, the relatively ‘pure’ cerebellar SCA, can present as a levodopa-responsive dystonia [54]. MRI showed cerebellar atrophy with normal-appearing basal ganglia, and the DAT SPECT revealed a reduced striatal dopamine reuptake [54]. On the contrary, there are several reports of inadequate levodopa response in the same SCAs (SCA 2,3, and 6) as well, although dopamine transporter imaging was not available for every patient [28,41,42,55,127]. The painful dystonia in the SCA3 patient with basal ganglia degeneration on MRI did not improve with levodopa [124]. Moreover, a patient of SCA21 who manifested cervical and upper limb dystonia with dystonic tremor had a normal brain MRI and DAT-SPECT, and showed poor response to levodopa [99]. In addition to the basal ganglia, the neuropathology of SCA (such as SCA 1, 2, and 3) also includes the thalamic nuclei [116]. In this regard, MRI volumetry has documented distinctive thalamic atrophy in SCA3 patients with dystonia [117]. Deep-brain stimulation surgery has been used in selected SCA patients with medication-refractory dystonia. The GPi was the commonest target, and showed benefit in some of them [12,29,124,128], although sometimes the effect was partial or transient [129,130]. The thalamus was also targeted, such as the ventral oral (Vo) and ventral intermediate (Vim) nuclei, which improved the dystonia and dystonic tremor in SCA patients at least initially [105,124,131].

Dysfunction of the cortico-striato-pallido-thalamo-cortical circuit (CSPTC) is evident in the different types of dystonia. The classical model of the striato-pallidal direct and indirect pathways is now integrated with the concept of the striatal matrix and striosome compartments [132]. The relationship of dopamine with dystonia appears complex. A dopaminergic excess would increase the direct pathway activity over the indirect pathway, thus minimizing the surround inhibition, and leading to the hyperkinetic state of dystonia. However, some of the dystonias may improve dramatically with levodopa. This may be explained by a selective decrease in the D1-receptor-mediated stimulation of the striosomal medium spiny projection neurons (MSNs) compared to the matrix in the milieu of a striatal dopamine deficiency state, which subsequently reduces the striosomal inhibitory output to the SNc, leading to a relative predominance of the direct pathway activity in the matrix [120]. It is possible that the effect of levodopa is further modified by the D2 receptor status, as a postsynaptic dysfunction may lead to an insufficient therapeutic response. Moreover, a lack of levodopa efficacy might also indicate a striatal dysfunction without significant nigrostriatal degeneration [99]. Importantly, the various thalamic nuclei act as components of the different circuits connecting the basal ganglia, cerebellum, and sensorimotor cortices (such as the ventral oral (Vo), ventral intermediate (Vim), and intralaminar nuclei corresponding to the CSPTC, CTC, and the direct cerebello-striatal pathways, respectively) [133]. Hence, involvement of the thalamus is also plausible in the dystonia pathophysiology in SCA.

### Cerebellar dysfunction

SCA is defined by cerebellar dysfunction. In the patient of SCA3 with dystonia discussed previously, the cerebellar hemispheres showed hypometabolism on FDG-PET, with normal cerebral cortical metabolism and striatal DAT scan [119]. The cerebellar hypometabolism improved after deep-brain stimulation of the Gpi and dentate nucleus. Tremor is common in SCA12, which is often dystonic. Despite the presence of dystonia, striatal atrophy is not evident in SCA12; rather, it consists of cerebellar and cerebral cortical atrophy [67]. The dopamine transporter scan was normal in a SCA12 patient with dystonia [71]. Cerebellar atrophy is the common finding in SCA14, presenting with dystonia, including a patient with writer’s cramp [74,76,77]. Although the MRI of a SCA14 patient with dystonic tremor showed globus pallidus iron accumulation (with normal DAT scan) [129], other reports did not reveal any basal ganglia atrophy [74,76,77]. Interestingly, in a SCA2 family of levodopa-responsive parkinsonism, one patient developed a lingual and jaw-opening oromandibular dystonia which did not respond to levodopa and pramipexole despite having a reduced DAT uptake [25]. This suggests that the dystonia might have another neural substrate.

The cerebello-thalamo-cortical (CTC) pathway (or the dentato-rubro-thalamic tract) is the primary output pathway of the cerebellum, and is implicated in tremor and dystonia due to its modulation of the various nodes connected in this network [67,133]. Reduced CTC activity was associated with a loss of inhibition in the supplementary motor area and the parietal cortex [120,134]. Recently, a direct cerebellar output to the basal ganglia has been identified. The dentate nuclei project to the striatal cholinergic interneurons via the thalamic intralaminar nuclei, and these interneurons modulate both the direct and indirect basal ganglia pathways, along with the corticostriatal synaptic plasticity [132,133]. An abnormal network activity in this cerebello-striatal pathway may alter the functioning of the cholinergic interneurons and disrupt the balance between the direct and indirect pathways, manifesting as dystonia. In addition to these connections, the cerebellum plays an important role in generating an appropriate internal model of movement, which is impaired in dystonia [121,122]. Also, the integration of the proprioceptive sensory information into the internal movement model could be impaired in cerebellar dysfunction [121]. This may influence the sensorimotor integration and maladaptive plasticity seen in focal task-specific dystonia.

**Figure 4:**
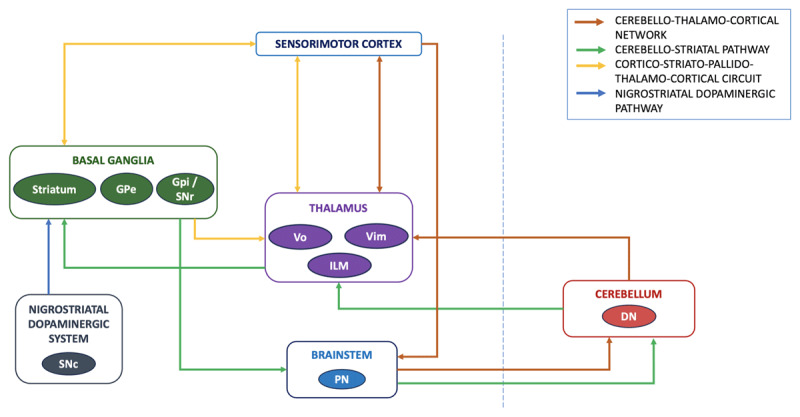
Networks involved in the pathophysiology of dystonia in Spinocerebellar ataxia. DN- Dentate nucleus, GPe- Globus pallidus externus, GPi- Globus pallidus internus, ILM- Intralaminar nuclei, PN- Pontine nuclei, SNc – Substantia nigra pars compacta, SNr – Substantia nigra pars reticulata, Vim – Ventral intermediate nucleus, Vo – Ventral oral nucleus.

In the presence of a combined dysfunction of the cerebellum, basal ganglia, nigrostriatal dopaminergic system, thalamus, and sensorimotor cortices, it is difficult to assess the extent of contribution of the cerebellum individually in producing the dystonia in SCA (Figure 4). However, evidence from SCA cases with a predominant cerebellar dysfunction suggests that the cerebellum is a key factor in the dystonia pathophysiology. Further studies utilizing advanced neuroimaging and neurophysiological techniques are required to determine whether the cerebellum is sufficient by itself to produce dystonia by modulating the various neural networks.

## Treatment of Dystonia in Spinocerebellar Ataxia

### Medical management

**Figure 5:**
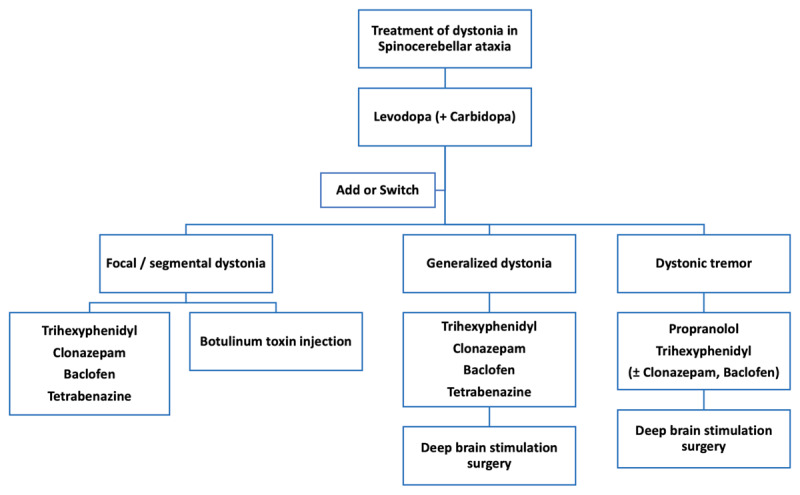
Treatment of dystonia in Spinocerebellar ataxia.

Medications used to treat dystonia in general have also been utilized in SCA patients. These include trihexyphenidyl (anticholinergic), clonazepam (benzodiazepine), baclofen, levodopa (with carbidopa/benserazide) and tetrabenazine (Figure 5). The response to levodopa is variable in SCA. Some of the patients showed remarkable improvement [23,46,125], even presenting as a levodopa-responsive dystonia [45,54], although others demonstrated minimal or no clinical benefit [28,41,42,55,127]. While the upper limb dystonia in a SCA6 patient decreased with a low dose of levodopa/carbidopa (100/10 mg twice daily) [54], other reports suggest efficacy at higher doses ranging from 300–600 mg/day [23,46,117]. Trihexyphenidyl reduced the dystonia in some patients when used alone [72], or in combination with baclofen [9,11], clonazepam [77], or levodopa [23]. However, the response was often partial, and several patients showed no improvement. Propranolol was used to treat the dystonic tremor with some benefit in SCA12 [67] and SCA21 [99], but showed no response in SCA35 (along with levodopa, primidone, trihexyphenidyl, gabapentin, and topiramate) [105]. While rehabilitation such as physical therapy, speech and voice therapy, and occupational therapy is discussed in ataxia and dystonia [135,136], specific studies on its role in dystonia in SCA are scarce.

### Botulinum toxin injection

Botulinum toxin (BoNT) injection has been used to treat focal/segmental dystonia in SCA. Retrocollis in a SCA1 patient improved with sequentially higher doses of BoNT type A injections [11]. There are several other reports of clinical benefit from BoNT injection in cervical [2,21,42,90,106], oromandibular [71,110], and limb dystonia (including writer’s cramp) [43,55,74,90,99] in the various SCAs such as SCA 1,2,3,6,12,14,17,21,35 and 36. However, in a series of SCA3 patients, significant adverse effects developed following BoNT injection, including a severe worsening of dysphagia after injecting for cervical dystonia [117]. Interestingly, two such patients in the series had chronic denervation changes in the limb muscles on electromyography [117]. BoNT was ineffective in a SCA3 patient with truncal dystonia [50], and another patient with limb dystonia [124].

### Surgical management

#### Deep brain stimulation surgery

Deep brain stimulation surgery (DBS) has been utilized in SCA patients with medication-refractory dystonia (Table 2). DBS of bilateral globus pallidus internus (GPi) produced significant resolution of dystonia in a SCA1 patient [12]. A left GPi DBS was beneficial for a SCA2 patient manifesting right upper limb dystonia [29]. In another SCA2 patient, cervical dystonia and tremor of the head, trunk and upper limbs improved with a subthalamic-thalamic DBS [131]. However, GPi followed by subthalamic nucleus (STN) DBS failed to produce any benefit in a SCA2 patient with generalised dystonia [28]. A series of three SCA3 patients treated with DBS for dystonia noted a mixed outcome with recurrence of the dystonia after an initial improvement [124]. Other patients of SCA3 have shown either a partial improvement [28,119], or a reappearance of dystonia [130], or no meaningful improvement [42,124]. GPi DBS produced a sustained benefit of dystonia and tremor lasting for more than 10 years in SCA17 [128], and partially alleviated the dystonic tremor of SCA14 [129]. GPi DBS improved upper limb dystonic tremor in a SCA17 patient without improvement of lower limb dystonia [135]. Vim DBS produced an ill-sustained reduction of the dystonic hand tremor in SCA35 [105]. A patient of SCA27A had substantial improvement of hand tremor with bilateral STN DBS [136]. Interestingly, later on, she developed a dystonia of the right hand and was treated with BoNT injection.

**Table 2:** Deep brain stimulation for dystonia in Spinocerebellar ataxia.


STUDY (AUTHOR AND YEAR)	SCA TYPE	PATIENT CHARACTERISTICS	DBS TARGET	OUTCOME

Copeland BJ et al., 2014 [12]	SCA1	• Craniocervical dystonia that was responsive to botulinum toxin injection • Progressed to the upper limbs and trunk involved • Baclofen, clonazepam not useful	Bilateral GPi	• Improvement of the dystonia • Right GPi- contacts 11–, 10+ with amplitude 2.2 V, pulse width 270 msec, and frequency 160 Hz • Left GPi- contacts 3–, 1+ with amplitude 2.0 V, pulse width 270 msec, and frequency 160 Hz • Amplitude increased over the first few month • Displayed some residual dystonia of the trapezius muscles and shoulders bilaterally that responded to botulinum toxin injection

Cheng N et al., 2018 [29]	SCA2	• Initial symptom was difficulty writing with the right hand • Writer’s dystonia progressed to other activity of right hand	Left GPi	• Significant improvement in the dystonia • Monopolar device achieved optimal settings at- lead C + 0–, frequency of 165 Hz, voltage of 2.4 V and pulse width of 450 μs

Freund HJ et al., 2007 [131]	SCA2	• Tremor of the arms, head and trunk • Torticollis	Subthalamic-thalamic (Vim, Vop, zona incerta and cerebello-thalamic projection)	• Stimulation was turned on at the third postoperative day • Tremor and torticollis significantly improved

Cui Z et al., 2023 [119]	SCA3	• Cerebellar ataxia and dystonia • Slight bilateral hand tremor, balance disorder, and gait abnormality • Neck stiffness and facial dystonia	GPi, dentate nucleus	• Improvement was 42% in SARA, 30% in BFMDRS movement, and 12.5% in BFMDRS disability score • Patient was not satisfied with the improved symptoms or their quality of life. This eventually led to the patient’s request to remove the leads and abandon the implantation of the pulse generator

Ikezawa J et al., 2023 [124]	SCA3	• Dystonia of the left lower limb • Dysarthria, oculomotor disturbances, and ataxia of the limbs and trunk additionally appeared, and had difficulty walking • Tizanidine, clonazepam, and botulinum toxin treatments were attempted but were ineffective	Right Vo, bilateral GPi	• DBS was performed on the right thalamic Vo nucleus for dystonia in the left upper and lower limbs • Upper and lower limbs markedly improved adequately to allow fine movement, such as holding a teacup • The pain greatly diminished as the dystonia symptoms improved • However, cerebellar ataxia remained unchanged • Stimulation effect did not persist in the long term. From the second year after surgery, dystonia in the left upper and lower limbs and cerebellar symptoms gradually worsened, and the pain, which had subsided, became more severe • Bilateral GPi DBS was added • 50-Hz frequency was the best to improve dystonia symptoms and it was well tolerated • 6 months later, painful dystonia in the left hand recurred, which did not improve despite various stimulation adjustments

		• Painful dystonia in the left lower limb, progressed to painful dystonia in the right and left upper limbs • Levodopa, diazepam and other medications without success	Bilateral GPi	• No significant changes, except for a slight improvement in pain in the upper and lower limbs, were observed

		• Left leg began to turn inwards only while walking • Painful dystonia was evident not only in the lower extremities but also in both the upper extremities	Bilateral GPi	• Patient was able to stand with assistance 1-month post-surgery owing to improvement in the apical foot position • 2 years postoperatively, painful dystonia in the left leg began to appear, which did not significantly improve despite stimulation adjustments

Muglan JA et al., 2016 [42]	SCA3	• Pronounced anterocollis associated with right tilt and twist and minimal sagittal and lateral shift and intermittent dystonic tremor • There was fixed and painful dystonia with no change with posture	GPi	• Did not improve the condition

Aupy J et al., 2018 [130]	SCA3	• Presented with cervical dystonia • Progressed to generalized dystonia involving the four limbs, the trunk and the face	Bilateral GPi	• Right lead, contacts 5 and 6, pulse width 210 μs, frequency 130 Hz, amplitude 2 V • Left lead, contacts 1 and 2, pulse width 210 μs, frequency 130 Hz, and amplitude 2.6 V • Patient was able to stand up without help and his swallowing improved • Cerebellar ataxia did not improve • Painful generalized dystonia progressively reccurred despite changes to the stimulation settings

Beaulieu-Boire I et al., 2016 [28]	SCA2	• Generalised dystonia (predominantly axial and right upper limb) • Inadequate response to baclofen, benzodiazepine, BoNT, levodopa, tetrabenazine, trihexyphenidyl	Bilateral STN, GPi	• No significant improvement

	SCA3	• Segmental dystonia (predominantly cervical)	Bilateral GPi	• Some improvement

Riso V et al., 2020 [129]	SCA14	• Trunk and limb tremor • Dystonic tremor with marked axial involvement	GPi	• Partially effective on tremor

Wagle Shukla A et al., 2023 [128]	SCA17	• Generalized dystonia and focal arm tremor	Bilateral GPi	• After surgery frequency 130 Hz, then reduced to 60 Hz • Improvement in dystonia, tremor • At 13 years of follow-up, although the ataxia has continued to worsen, DBS therapy has led to persistent improvements in dystonia, tremor

Oyama G et al., 2014 [138]	SCA17	• Generalized dystonia, with a severe inversion of his left foot • Progressed to include a twisting of his neck and shoulders	Bilateral GPi	• Improvement of his upper extremity dystonic tremor • No improvement noted in his lower extremity dystonia • Dystonia scales continued to worsen at his 12-month follow-up and multiple DBS settings were attempted without dystonia and ataxia benefit

Keller Sarmiento IJ et al., 2025 [135]	SCA27A	• 58-year-old right-handed female patient • At age 3 years a hand tremor was noticed. Her gait became unstable at around the age of 33 years • At age 42 years she underwent bilateral STN-DBS	Bilateral STN	• Significant improvement in tremor • Over time, she developed right-hand dystonia, which was treated with botulinum toxin injection

Fasano A et al., 2017 [105]	SCA35	• Dystonic hand tremor • Disabling action tremor of both upper extremities	Right Vim	• Initial improvement of tremor was not sustained


BFMDRS- Burke-Fahn-Marsden Dystonia Rating Scale, DBS- Deep Brain Stimulation, GPi- Globus Pallidus internus, SARA- Scale for the Assessment and Rating of Ataxia, SCA- Spinocerebellar Ataxia, STN- Subthalamic Nucleus, Vim- Ventral intermediate nucleus of thalamus, Vo- Ventral oral nucleus of thalamus.

#### Magnetic resonance-guided focused ultrasound

Recently, magnetic resonance-guided focused ultrasound (MRgFUS) was found effective in dystonic tremor (unilateral thalamotomy), focal hand dystonia (ventro-oral thalamotomy), and cervical dystonia (unilateral pallidothalamic tractotomy) [137]. Mild gait ataxia may be an adverse effect of MRgFUS. A patient of SCA12 manifesting severe upper limb action tremor, underwent unilateral MRgFUS targeting the cerebello-thalamo-cortical tract [138]. His right arm tremor improved significantly, and the effect persisted after 11 months. There was gait impairment in the post-operative period, but it resolved within 3 months [138].

## Conclusion

Dystonia is an important and underrecognized non-ataxic manifestation of SCA, occurring across multiple genetic subtypes with variable frequency and phenotype. Although most commonly reported in SCA3 and SCA2, it is also documented in SCA1, SCA6, SCA12, SCA14, SCA17, and several rarer forms such as SCA21 and SCA48. Clinical presentations range from focal dystonia (cervical dystonia, writer’s cramp, blepharospasm, lingual or oromandibular dystonia) to segmental, multifocal, and generalized dystonia. In many patients, dystonia accompanies cerebellar ataxia, but it may precede ataxia by years and even be the dominant presenting symptom. The pathophysiology involves the interconnected circuits, including the cerebellum and other subcortical and cortical structures such as the basal ganglia, thalamus, nigrostriatal dopaminergic pathways, and sensorimotor cortex. These interactions likely contribute to the loss of inhibition, maladaptive plasticity, and abnormal sensorimotor integration. Variable responsiveness to therapy further suggests heterogeneity in the underlying mechanisms. Management is symptomatic and individualized. Medications used include levodopa, trihexyphenidyl, clonazepam, baclofen, and tetrabenazine, with variable efficacy. Botulinum toxin is useful for focal or segmental dystonia, particularly cervical, limb, and oromandibular dystonia. In refractory cases, deep brain stimulation, most commonly targeting the GPi, has shown benefit in selected patients, though outcomes are mixed. Overall, recognizing dystonia in SCA is clinically important because it may aid in the diagnosis, and offers treatable targets to improve function and quality of life.

## Additional File

The additional file for this article can be found as follows:

10.5334/tohm.1163.s1 Supplementary Table. Characteristic features of Dystonia in each Spinocerebellar Ataxia type.
