# Supplementary material for: Spectrum of Dystonia in Spinocerebellar Ataxia
Source: Tremor Other Hyperkinet Mov (N Y). 2026 May 29;16:35. doi: 10.5334/tohm.1163 (PMC13220735; doi:10.5334/tohm.1163)
Supplement: Supplementary Table. — Characteristic features of Dystonia in each Spinocerebellar Ataxia type. [file tohm-16-1-1163-s1.pdf]

Supplementary Table: Characteristic features of Dystonia in each Spinocerebellar Ataxia type

SCA Type	Study (Author and Year)	Characteristic features of dystonia in each SCA type
SCA1	• Khwaja GA et al., 2016 [9]	• Presentation as Task specific dystonia- Writer's cramp
	• Kikuchi A et al., 2016 [10]	• Good response to Botulinum toxin therapy
	• Copeland BJ et al., 2014 [11]	• Generalised Dystonia and recurrent dystonic crisis with good response to Bilateral GPi DBS
SCA2	• Radhakrishnan DM et al., 2018 [15]<br>• Boesch SM et al., 2007 [19]<br>• Zárubová K et al., 2006 [20]	• Cervical dystonia- most common dystonia presentation in SCA2
	• Furtado S et al., 2004 [30]<br>• Sun YM et al., 2023 [31]	• Dystonia-parkinsonism phenotype
	• Walsh R et al., 2009 [25]	• Rarely Spasmodic Dysphonia
	• Avelino MA et al., 2014 [32]	• Early neonatal onset Dystonia
	• Cheng N et al., 2018 [28]	• Task specific dystonia- Writer's cramp
SCA3	• Méndez-Guerrero A et al., 2018 [40]	• Task specific writer's cramp
	• Chen SJ et al., 2019 [14]<br>• Catai LMP et al., 2018 [39]	• Dystonia as a relatively common manifestation
	• Zhang XL et al., 2021 [43]	• Dopamine responsive dystonia

	• Wilder-Smith E et al., 2003 [44]<br>• Nandagopal R et al., 2004 [45]	
	• Mitchell N et al., 2019 [48]	• Early onset tongue dystonia
	• Vasconcellos LF et al., 2014 [49]	• Severe truncal dystonia
SCA6	• Ikezawa J et al., 2023 [103]	• Levodopa responsive dystonia
	• Muzaimi MB et al., 2003 [105]<br>• Olszewska DA et al., 2015 [106]	• Task specific writer's cramp
SCA8	• Ushe M et al., 2012 [51]	• Oromandibular dystonia
	• Koutsis G et al., 2012 [52]	• Limb dystonia
SCA10	• Gatto EM et al., 2007 [53]	• Cervical dystonia
SCA11	• Giunti P et al., 2012 [110]	• Cervical dystonia (laterocollis)
SCA12	• Ganos C et al., 2014 [56]	• Spasmodic dysphonia
	• Neo S et al., 2024 [57]	• Feeding dystonia
	• O'Hearn E et al., 2001 [59]	• Early childhood onset reported
SCA14	• Ito M et al., 2024 [63]<br>• Erro R et al., 2014 [64]	• Task specific writer's cramp eveb prior to ataxia
	• Foncke EM et al., 2010 [67]	• Dystonia-myoclonus<br>• Dystonic trunk tremor
SCA17	• Loy CT et al., 2005 [72]<br>• Schneider SA et al., 2006 [78]	• Dystonia-chorea

SCA19/22	• Kurihara M et al., 2018 [80]	• Dystonia and myoclonus
	• Pollini L et al., 2020 [81]	• Oromandibular dystonia and dystonic head tremor
SCA21	• Yahya V et al., 2024 [85]	• Task specific writer's cramp with dystonic tremor
	• Sorrentino U et al., 2024 [86]<br>• Cherian A et al., 2022 [87]	• Myoclonus-dystonia
SCA28	• Reyes NGD et al., 2023 [89]	• Generalised dystonia with Spasmodic dysphonia
SCA35	• Fasano A et al., 2017 [91]	• Dystonic head tremor
	• Thirumurugesan SL et al., 2022 [92]<br>• Wang JL et al., 2010 [93]	• Cervical dystonia
SCA36	• Baviera-Muñoz R et al., 2023 [94]<br>• Nakazato Y et al., 2015 [95]	• Cervical dystonia and dystonic tremor
	• Miyashiro A et al., 2013 [96]	• Jaw opening dystonia and feeding difficulty
SCA48	• De Michele G et al., 2019 [97]<br>• Lieto M et al., 2020 [98]	• Chorea-dystonia
SCA49	• Garg D et al., 2024 [101]	• Cervical dystonia with dystonic head tremor
SCA50	• Dalla Zanna G et al., 2025	• Appendicular dystonia

DBS- Deep Brain Stimulation, GPi- Globus Pallidus internus, SCA- Spinocerebellar Ataxia
